# Oropharyngeal cancer: Lack of human papillomavirus awareness and economic burden in the United States

**DOI:** 10.1002/ctm2.70062

**Published:** 2024-11-18

**Authors:** Matt Lechner, Liam Masterson, Shiri Mermelstein, Jacklyn Liu, Umar Rehman, Michelle Chen, James O'Mahoney, F. Christopher Holsinger

**Affiliations:** ^1^ Division of Surgery and Interventional Science University College London London UK; ^2^ Cancer Institute, University College London London UK; ^3^ Department of ENT Cambridge University Hospitals NHS Trust Cambridge UK; ^4^ Centre for Health Policy and Management Trinity College Dublin Ireland; ^5^ Department of Otolaryngology‐Head and Neck Surgery Stanford University School of Medicine Palo Alto California USA

**Keywords:** awareness, broader societal costs, economy, head and neck cancer, HPV‐associated oropharyngeal cancer

1

Both in the United States and the United Kingdom, with similar trends in continental Europe, the rates of male HPV‐associated oropharyngeal squamous cell carcinoma (OPSCC) have overtaken the incidence rates of cervical cancer in women. Persistent, oropharyngeal human papillomavirus (HPV) infection has been the major driver behind this rapid increase.^1^ Despite these alarming numbers, the awareness of HPV has been extremely low, which may have implications on vaccine uptake. We conducted a survey of 4871 adults across the United States to ascertain the current levels of HPV awareness in relation to oropharyngeal cancer. The cohort was 49.9% female and 49.7% male, with patient age well distributed across the various age groups (see Supplemental Table 1). Regarding OPSCC risk, 43.3% and 78.5% identified alcohol and smoking as risk factors, respectively. 72.8%, 37.7%, 30.3% and 42.3% identified chewing tobacco, betel leaf, catechu/areca nut and marijuana use as additional risk factors, respectively.

Crucially, only one‐third (31.3%) were aware that HPV is one of the most important risk factors of OPSCC. Indeed, only 62.3% of respondents had heard of HPV prior to taking the survey. Of these, 68.5% were aware that HPV can be sexually transmitted and 60.0% knew that it can be transmitted through oral sex. Of the entire cohort, less than two‐thirds (62.7%) were aware that there is a vaccine against HPV.

Altogether, these results show that the awareness of HPV and its link to OPSCC is still limited in the United States, despite the introduction of gender‐neutral vaccination and amid rapidly increasing rates of disease.

Indeed, it is expected that OPSCC rates will continue to rise over the next two decades. Several authors have attempted to assess the costs of OPSCC in the United States, using data from the early 2000s or based on a state‐specific database. The most recent estimate of oropharyngeal cancer treatment cost in the United States was published by Wu et al.,^2^ who used a large nationwide database (Truven MarketScan Commercial Claims and Encounter Database). They estimated that the average total lifetime treatment cost per newly diagnosed commercially insured patient would be $152 378 in 2015 prices, equivalent to $178 010 in 2020 prices (Medical Care Consumer Price Index, available from the US Bureau of Labor Statistics, 2020). The overall cost for OPSCC treatment in the United States was estimated by combining Wu et al.^2^ treatment cost for commercially insured patients with the incidence rates of OPC^3^ and the population projections (US Bureau, 2020), which gave a total direct cost of over $4.2 billion in 2020.

Broader societal costs of OPSCC were based on Pearce et al.^4^ assessment of lost workplace productivity cost due to head and neck cancer in Ireland, equivalent to $311 926 per case diagnosed (in 2020 prices and after a proportionate adjustment to account for the relative difference in GDP per capita in the United States and Ireland). While there have been few analyses of the productivity loss due to OPSCC in the United States, these assessments used old data. The Pearce et al.^4^ estimates account for the proportion of patients that return to work following treatment. According to SEER 2013–2017 data, 53.5% of patients diagnosed with OPSCC were of working age (younger than 65 years old). Applying the above loss productivity cost to the estimated incidence of oropharyngeal cancer (under age 65) in the United States in 2020 yielded an overall cost of over $3.95B in 2020.

Given that approximately 70% of OPSCC cases in the United States are attributed to HPV (CDC, 2020b), the overall treatment cost for HPV‐positive OPSCC patients is estimated at $2.95B, and the overall societal cost is estimated at nearly $2.77B in 2020. It is anticipated that more than 446 139 people will be diagnosed with HPV‐positive OPSCC from 2020 to 2039, based on the projected population and annual incidence. Combining the projected annual number of new cases with the estimated direct and broader societal costs yields a discounted total of $114.7B over the period between 2020 and 2039 (Supplemental Figure 1). The future costs were discounted at 3% per annum to yield a present value of this estimate over the projected period (Supplemental Figure 2).^5^

In conclusion, this data is alarming both regarding the immense costs associated with the rapidly increasing rates of HPV‐associated oropharyngeal cancer and regarding the lack of awareness of this problem amongst the US population. While HPV vaccination will protect the next generation of patients, a risk‐stratified screening program is urgently needed to reverse this trend, associated morbidity and mortality and the significant burden on healthcare systems and society.

## AUTHOR CONTRIBUTIONS

*Conceptualization*: Matt Lechner, Liam Masterson, F. Christopher Holsinger, and Michelle Chen; *Methodology*: James O'Mahoney, Shiri Mermelstein, Matt Lechner, Liam Masterson, F. Christopher Holsinger, and Michelle Chen; *Formal analysis and investigation*: James O'Mahoney, Shiri Mermelstein, Matt Lechner, Liam Masterson, F. Christopher Holsinger, Jacklyn Liu, Umar Rehman, and Michelle Chen; *Writing—original draft preparation*: James O'Mahoney, Shiri Mermelstein, Matt Lechner, Liam Masterson, F. Christopher Holsinger, Jacklyn Liu, Umar Rehman, and Michelle Chen; *Supervision*: Matt Lechner, Liam Masterson, F. Christopher Holsinger, and Michelle Chen.

## ETHICS STATEMENT

Ethical approval was not required, as the recruitment involved non‐vulnerable adults (aged 18 or over) who were able to provide informed consent to participate in the study.

## Supporting information

Supporting Information
