# Supplementary material for: Oropharyngeal cancer: Lack of human papillomavirus awareness and economic burden in the United States
Source: Clin Transl Med. 2024 Nov 18;14(11):e70062. doi: 10.1002/ctm2.70062 (PMC11573731; doi:10.1002/ctm2.70062)
Supplement: Supplementary file 1 — Supporting Information [file CTM2-14-e70062-s001.docx]

**Supplemental Material**

Supplemental Figures:

**Supplemental Figure 1:** Projected Overall Cost of OPSCC in the US (Undiscounted), 2020-2039 (based on SEER18 age-adjusted incidence trends (linear), not included in the text doc)

**Supplemental Figure 2:** Projected Overall Cost of OPSCC in the US (Discounted), 2020-2039

**Supplemental Table**

| Age Group | Frequency | Percentage |
| --- | --- | --- |
| 18-24 | 198 | 4.1 |
| 25-34 | 904 | 18.6 |
| 35-44 | 646 | 13.3 |
| 45-54 | 754 | 15.5 |
| 55-64 | 600 | 12.3 |
| 65-74 | 835 | 17.1 |
| 75 or older | 597 | 12.3 |
| Missing | 337 | 6.9 |
| Total | 4871 | 100 |

**Supplemental Table 1**. Distribution of Respondents by Age Group.
